# Amino acid T25 in the substrate-binding domain of SARS-CoV-2 nsp5 is involved in viral replication in the mouse lung

**DOI:** 10.1371/journal.pone.0312800

**Published:** 2024-12-06

**Authors:** Yoshiro Sugiura, Kenta Shimizu, Tatsuki Takahashi, Shiori Ueno, Haruka Tanigou, Sodbayasgalan Amarbayasgalan, Wataru Kamitani

**Affiliations:** Department of Infectious Disease and Host Defense, Graduate School of Medicine, Gunma University, Gunma, Japan; National Center of Biotechnology (CNB-CSIC), SPAIN

## Abstract

Severe acute respiratory syndrome coronavirus 2 (SARS-CoV-2) non-structural protein 5 (nsp5) is a cysteine protease involved in viral replication and suppression of the host immune system. The substrate-binding domain of nsp5 is important for its protease activity. However, the relationship between nsp5 protease activity and viral replication remains unclear. We confirmed the importance of amino acid T25 in the nsp5 substrate-binding domain for viral replication using a split luciferase assay. By generating recombinant viruses using bacterial artificial chromosomes, we found that the proliferation of viruses with the T25I mutation in nsp5 was cell-dependent in culture. Furthermore, mice infected with the T25I mutant recombinant virus with a mouse acclimation backbone showed weight loss and increased lung viral load, similar to the wild-type (WT) infected group, up to 3 days after infection. However, after day 4, the lung viral load was significantly reduced in the T25I-infected group compared to that in the WT-infected group. This suggests that nsp5 T25 is involved in the pathogenesis of SARS-CoV-2.

## Introduction

Human coronaviruses primarily cause respiratory and digestive diseases. Severe acute respiratory syndrome coronavirus 2 (SARS-CoV-2) is the causative agent of coronavirus disease 2019 (COVID-19), which is the most recent pandemic [1–4]. Approximately two-thirds of the coronavirus genome consists of the ORF1ab region, which encodes 16 non-structural proteins (NSPs). SARS-CoV-2, SARS-CoV-1, and MERS-CoV have two proteases in the ORF1ab region, namely, nsp3 and nsp5. nsp3 acts as a papain-like protease and nsp5 acts as a 3C-like protease. These two proteases are necessary for the production of mature NSPs in the early stages of coronavirus replication [5–8].

nsp5 is a cysteine protease in the chymotrypsin family that primarily cleaves peptides at P2-P1-P1’ residues consisting of leucine-glutamine-alanine/serine. The enzymatic activity of nsp5 is conserved among coronaviruses; in fact, recombinant mouse hepatitis virus was successfully generated with nsp5 from human coronaviruses OC43 and HKU1 [9]. This suggests that the targeted inhibitors of nsp5 are broadly effective against coronaviruses. Therefore, it is important to develop inhibitors that target the protease activity of nsp5. Several protease inhibitors have been reported to be effective against nsp5 [10, 11].

SARS-CoV-2 nsp5 comprises 306 amino acids and three domains. Domains I and II form a chymotrypsin-like fold and domain III comprises a cluster of five alpha helices connected to domain II by a long loop [12, 13]. Nsp5, also called Mpro or 3CL protease, is a three-domain cysteine protease with catalytic amino acids C145 and H41 located in the cleft between domains I and II [14]. Inhibiting Mpro in SARS-CoV-2 is a promising strategy for developing oral antiviral therapies for COVID-19 [15]. The oral antiviral drug, Nirmatrelvir, which targets the nsp5 protease of SARS-CoV-2, has been clinically effective reducing the risk of severe COVID-19 progression by approximately 90% [16, 17]. Similarly, Ensitrelvir targets nsp5’s enzymatic activity and reduces disease progression [18]. However, concerns about viral resistance to drugs persist, with resistant viruses observed in cell culture experiments [19]. Therefore, it is important to elucidate the relationship between key amino acids for enzyme activity and viral growth in nsp5 using recombinant viruses.

SARS-CoV-2 nsp5 cleaves several host proteins, including histone deacetylase (HDAC), DCP1A, and NF-κB essential modulator (NEMO) [20–23]. NEMO is a key molecule in the RIG-I-dependent pathway [24]. Thus, nsp5 suppresses the production of type I interferons (IFNs) resulting from NEMO cleavage [20]. Interestingly, the cleavage of NEMO by nsp5 is also conserved in other coronaviruses, such as SARS-CoV-1, feline coronavirus, porcine epidemic diarrhea virus, and porcine delta coronavirus [20, 25–27]. The cleavage efficiency varies among viruses, and SARS-CoV-2 nsp5 has been reported to cleave NEMO more efficiently than SARS-CoV-1 nsp5 [20]. SARS-CoV-2 nsp5 interacts with HDAC2, which disrupts the interaction of HDAC2 with IRF3. The protease activity of nsp5 is required for the inhibition of IFNβ, IL-6, and IL-1β expression [21].

The activity of nsp5 is important for viral replication and suppression of immune system signaling in infected cells [28].

The enzymatic activity of nsp5 plays a crucial role in cleaving the ORF1ab polypeptide chain, which is essential for coronavirus replication. It is therefore necessary to elucidate the role of the amino acid residues of nsp5 in viral replication and virulence. Therefore, we performed a split-luciferase bioassay to investigate the protease activity of nsp5 and elucidate its effect on viral replication. Our study sheds light on the involvement of nsp5 T25 in the severity of pneumonia caused by SARS-CoV-2.

## Materials and methods

### Cell and viral cultures

Human embryonic kidney cells expressing human angiotensin-converting enzyme 2 (293T/hACE2) were generated using a lentiviral expression system. Cells expressing VeroE6-transmembrane serine protease 2 (VeroE6/hTMPRSS2) [29] were maintained in Dulbecco’s modified minimum essential medium (Nacalai Tesque, Kyoto, Japan) containing 10% heat-inactivated fetal bovine serum, 100 U/mL penicillin, and 100 μg/mL streptomycin (Nacalai Tesque). All the cells were cultured in a humid atmosphere containing 5% CO_2_ at 37°C.

Mouse-adapted SARS-CoV-2 QHmusX strain [30] was kindly provided by Dr. Nagata (National Institute of Infectious Diseases, Shinjuku, Tokyo, Japan). All experiments using SARS-CoV-2 were approved by the Institutional Biosafety Committee, and carried out in BSL-3 facilities.

### Plasmid DNA construction

SARS-CoV-2 nsp5 with a C-terminal FLAG tag was cloned into pCAGGS-MCS to generate pCAGGS-SARS2-nsp5-FLAG. Using pCAGGS-SARS2-nsp5-FLAG as a template, inverse PCR was used to generate variants with specific mutations: pCAGGS-SARS2-nsp5-T25I-FLAG, pCAGGS-SARS2-nsp5-T45I-FLAG, pCAGGS-SARS2-nsp5-S46F-FLAG, pCAGGS-SARS2-nsp5-D48N-FLAG, pCAGGS-SARS2-nsp5-M49I-FLAG, pCAGGS-SARS2-nsp5-L50F-FLAG, pCAGGS-SARS2-nsp5-V186F-FLAG, pCAGGS-SARS2-nsp5-R188K-FLAG, and pCAGGS-SARS2-nsp5-T190I-FLAG. An expression vector lacking nsp5 enzymatic activity was generated by creating pCAGGS-SARS2-nsp5-C145A-FLAG through inverse PCR, which introduced a mutation at residue C145.

pGlo-VTFQS was constructed to examine the protease activity of nsp5 in transfected cells using pGlo-30F-VRLQS [31] as a template. pGlo-VTFQS encoding *Photuris pennsylvanica* luciferase with the inserted amino acid sequence VTFQS is recognized and cleaved by the SARS-CoV-2 nsp5 protease 3CLpro. The pGlo-30F-VRLQS biosensor expression plasmid was a gift from Dr. Susan Baker (Loyola University, Chicago, IL, USA).

### Immunofluorescence assay and western blot analysis

293T cells were seeded into a Nun Lab-Tek Chamber Slide System at 2.0 × 10^5^ cells per well. After overnight incubation at 37°C, cells were transfected with 1.0 μg of pCAGGS nsp5 expression plasmid using TransIT-LT1 Transfection Reagent (Mirus Bio). Following incubation at 37°C, transfected cells were fixed with 10% Formaldehyde Neutral Buffer Solution for 10 min and permeabilized with phosphate-buffered saline containing 1% TritonX for 10 min. Anti-DDDDK (FLAG)-tag mAb (Medical & Biological Laboratories, Tokyo, Japan) was used as the primary antibody and goat anti-mouse IgG (H+L)-CF 488A (Sigma, St. Louis, MO) was used as the secondary antibody. Cells were examined using an EVOS M7000 imaging system (Thermo Fisher Scientific).

293T cells were seeded at a density of 4.0 × 10^5^ cells per well in Violamo 6-well plates. After overnight incubation at 37°C, cells were transfected with 2.0 μg of pCAGGS nsp5 expression plasmid using TransIT-LT1 Transfection Reagent (Mirus Bio). Following 48 h of incubation at 37°C, total proteins were extracted from transfected cells and subjected to western blot analysis as previously described [32]. Anti-DDDDK (FLAG)-tag mAb (MBL) and anti-actin antibody (Sigma) were used as primary antibodies, and goat anti-mouse IgG (H+L)-HRP (Santa Cruz Biotechnology, Santa Cruz, CA, USA) was used as the secondary antibody. ChemiLumi One Ultra (Nacalai Tesque) was used for visualization using a LuminoGraph I image analyzer system (ATTO, Tokyo, Japan).

### Luciferase assay

293T cells were seeded in 24 well-plates (Violamo) at a density of 1×10^5^ cells/well and cultured overnight. The seeded cells were transfected with pGlo-VTFQS and nsp5 expression plasmids using the TransIT-LT1 Transfection Reagent (Mirus Bio) according to the manufacturer’s instructions. pCAGGS-MCS, a backbone for the nsp5 expression plasmid, was used as a control. Transfected cells were cultured for 48 h and then lysed with Passive Buffer (Promega). Luciferase activity was measured using a GloMax Explorer System (Promega). Firefly luciferase activity was measured using a Luciferase Assay System (Promega). A Nano-Glo Luciferase Assay System (Promega) was used to measure the luciferase activity. Luciferase activity was normalized to the nanoluciferase activity. All experiments were performed in triplicates.

### BAC DNA construction and recombinant virus

We previously established a reverse genetics system for SARS-CoV-2 using the BAC system [32]. cDNA clones of the T25I mutation in nsp5 were constructed from pBAC-SARS2-QHmusX using a Red/ET recombination system counterselection BAC modification kit (Gene Bridges, Heidelberg, Germany). This construct was designated as pBAC-SARS2-QHmusX-T25I. Sequence analysis was performed by Eurofins Scientific (Tokyo, Japan) to confirm the substitutions. The rSARS2-QHmusX-WT and rSARS2-QHmusX-T25I viruses were recovered from transfected VeroR6/hTMPRSS2 cells.

### Real-time PCR

Total RNAs from the cells were extracted using a PureLink RNA Mini Kit (Thermo Fisher Scientific) according to manufacturer’s instructions and stored at −80°C until use. RNA expression of the SARS-CoV-2 N gene was determined using a Thunderbird one-step qPCR Probe Mix (TOYOBO), and the reaction was performed using a StepOne Real-Time PCR System (Applied Biosystems, Waltham, MA, USA). To quantify N RNA, we used the primer pair N_Sarbeco_F1 (5′-CACATTGGCACCCGCAATC-3′) and N_Sarbeco_R1 (5′-GAGGAACGAGAAGAGGCTTG-3′), and the FAM-labeled specific probe, N_Sarbeco_P1 (5′-ACTTCCTCAAGGAACAACATTGCCA-3′). Cycling conditions were 95°C for 1 min, followed by 40 cycles of 95°C for 15 s and 58°C for 45 s. The copy number of the N gene was calculated using in vitro-transcribed N RNA.

### Animal experiments

All the experiments were approved by the Gunma University Animal Care and Experimentation Committee. Female BALB/c mice (17-week-old) were obtained from SLC (Shizuoka, Japan). BALB/c mice were infected with 3×10^4^ or 5×10^4^ 50% tissue culture infectious dose (TCID_50_)/head of recombinant SARS-CoV-2 via the nasal route. Body weight was monitored daily for up to 4 days post-infection. Lung tissues were collected 4 days post-infection. The tissues were separated for real-time PCR and TCID_50_ assays. The following steps were taken to alleviate suffering: in all experimental procedures that involved the potential for pain or distress, the animals were anaesthetized using isoflurane. All procedures were performed under anesthesia with isoflurane, and animals were closely monitored during recovery. Signs of pain or distress were carefully observed, and any animal exhibiting such signs was provided with appropriate analgesia.

### Histopathological analysis

For histological analysis, the lung tissues were fixed in a 10% formaldehyde neutral buffer solution (Nacalai Tesque) and embedded in paraffin. Paraffin specimens were cut into 4-mm thick sections, which were subjected to hematoxylin and eosin (HE) staining. To detect the N protein of SARS-CoV-2 in lung tissues using immunohistochemistry, tissue sections were incubated with anti-SARS-CoV-2 N monoclonal antibody (R&D Systems, clone 1035111) and then assessed with the Histofine MAC-PO Multi kit (Nichirei Biosciences).

### Statistical analyses

To assess statistical significance, a two-way analysis of variance (ANOVA) was performed using GraphPad Prism ver. 10 (GraphPad Software, San Diego, CA, USA). Statistical significance was defined as a p value of < 0.05.

## Results

### Subcellular localization of nsp5 mutants in transfected cells

To understand the correlation between the protease activity of SARS-CoV-2 nsp5 and viral replication, we constructed an expression plasmid for nsp5 based on the information reported by Gao et al. regarding the binding domain of SARS-CoV-2 nsp5 [33].

**Fig 1:**
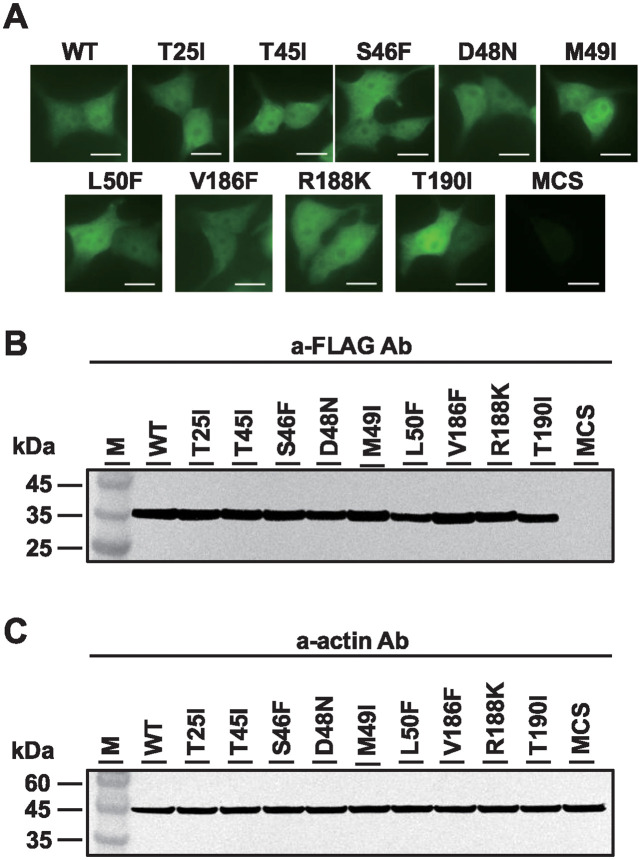
Subcellular localization of SARS-CoV-2 nsp5 and mutants. (A) 293T cells were transfected with nsp5 expression plasmids with FLAG. After 24 h, the subcellular localization of the expressed SARS-CoV-2 nsp5 was examined using anti-FLAG and anti-mouse IgG (H+L)-CF488 as primary and secondary antibodies, respectively. Scale bar = 10 mm. (B, C) Total intracellular proteins were extracted 24 post-transfection, and western blot analysis was performed using anti-FLAG and anti-beta actin antibodies. M represent molecular weight markers. pCAGGS-MCS DNA-transfected samples were used as controls.

293T cells transfected with wild-type (WT) or mutant SARS-CoV-2 nsp5 plasmids were subjected to immunofluorescence assay 24 h post-transfection. Both WT and mutant nsp5 proteins were observed in the nucleus and cytoplasm of transfected cells (Fig 1A), which is consistent with previous findings [7, 34]. Immunoblotting of lysates from mutant-transfected cells showed identical expression levels across all samples (Fig 1B and 1C). These results suggest that mutations in the binding region do not alter the subcellular localization of nsp5.

### Three nsp5 mutants showed reduced protease activity

**Fig 2:**
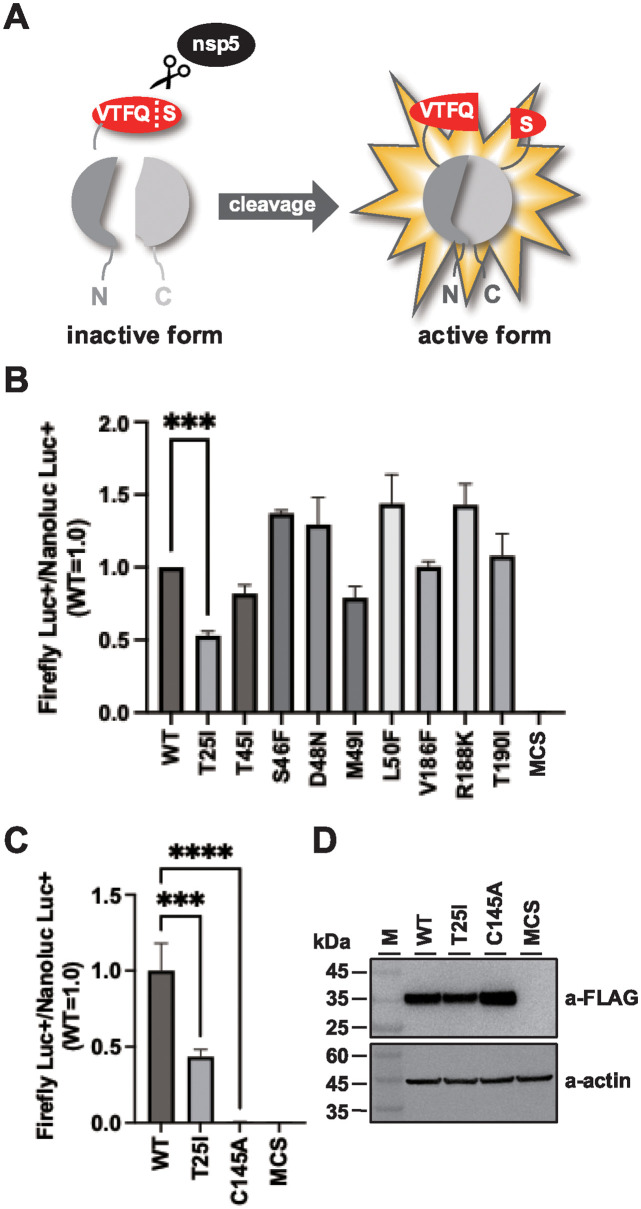
Split luciferase assay for the detection of SARS-CoV-2 nsp5 protease activity. (A) Schematic of the reporter assay to detect the protease activity of SARS-CoV-2 nsp5. The nsp5 cleavage sequence, VTFQS, was inserted between the luciferase genes; in the presence of nsp5, Q and S in the VTFQS sequence are cleaved and luciferase luminescence is detected. (B) nsp5 mutant protease activity detected using the split luciferase assay. 293T cells were transfected with nsp5 expression plasmids with pGlo-VTFQS and pNL1.1 (nanoluciferase-expressing plasmid). Intracellular luciferase activity was measured after 48 h. The luciferase activity in each sample was normalized to the nanoluciferase activity. The values represent the means ±SD derived from three independent experiments. ***P < 0.001. (C) 293T cells were transfected with nsp5-C145A expression plasmid along with pGlo-VTFQS and pNL1.1. Intracellular luciferase activity was measured after 48 h. Each sample’s luciferase activity was normalized against the nanoluciferase activity. The values represent the means ±SD from three independent experiments. ****P < 0.0001, ***P < 0.001. (D) Total intracellular proteins were isolated 24 post-transfection, and subjected to western blot analysis using anti-FLAG and anti-beta actin antibodies. M represents molecular weight markers.

We conducted a cell-based assay to examine nsp5 protease activity using a split-luciferase system. The nsp5 cleavage sequence VTFQS was inserted into the luciferase gene. Cells transfected with WT nsp5 had significantly increased luciferase activity compared to that in the control (Fig 2B). This result is consistent with those of previous reports [35, 36]. Although six mutants showed similar activity, three mutants (T25I, T45I, and M49I) exhibited reduced activity, with T25I showing a 50% decrease, indicating the importance of T25 in nsp5 enzymatic activity.

Subsequently, the extent to which the T25I mutation in nsp5 reduced enzyme activity was confirmed by creating nsp5-C145A, where the 145^th^ amino acid in the nsp5’s enzyme active site was changed from C to A [36]. Cells expressing C145A exhibited reduced luciferase activity levels comparable to those of the negative control (MCS) (Fig 2C). As shown in Fig 2D, nsp5-C145A expression in cells was comparable to that of WT and nsp5-T25. These findings suggested that the T25I mutation caused a reduction of approximately 50% in the enzyme activity of nsp5.

### Growth of a recombinant virus with T25I in nsp5 was cell-dependent

**Fig 3:**
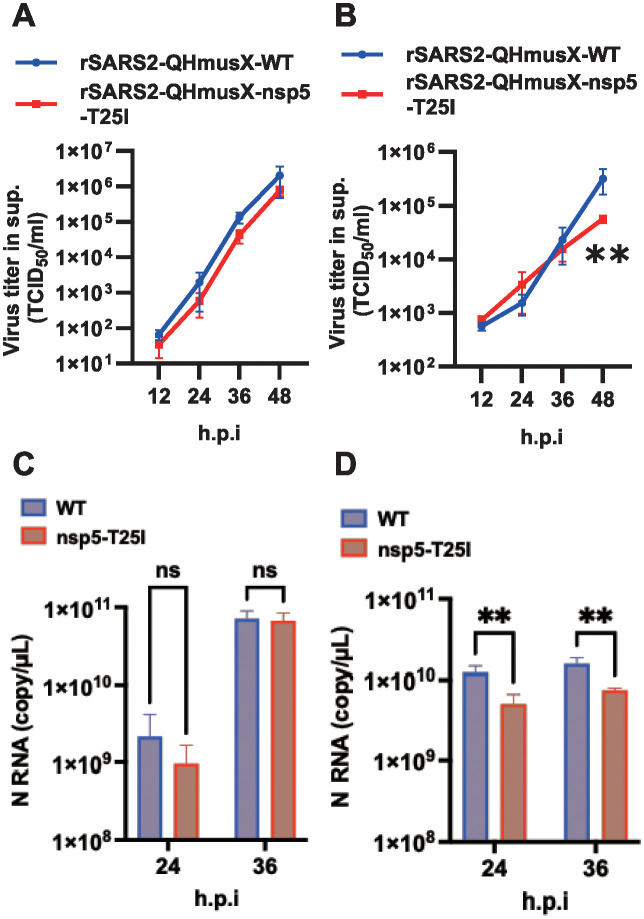
Effect of the nsp5 T25I mutation on virus production using a recombinant, mouse-adapted QHmusX strain. Growth kinetics of recombinant viruses in VeroE6/hTMPRSS2 cells (A) and 293T/hACE2 cells (B). VeroE6/hTMPRSS2 and 293T/hACE2 cells were inoculated with the recombinant viruses at MOI of 0.0001 and 0.01, respectively. Cells infected with rSARS2-QHmusX-WT or rSARS2-QHmusX-nsp5-T25I were cultured for the indicated time periods. Viral titers in the culture supernatants were determined using the TCID_50_ assay. The values represent the means ±SD from three independent experiments. **P < 0.01. Expression of N RNA from recombinant viruses in VeroE6/hTMPRSS2 cells (C) and 293T/hACE2 cells (D) measured using one-step real-time PCR. ns, no statistically significant difference; h.p.i., hours post-infection. The values represent the means ±SD from three independent experiments. **P < 0.01.

We constructed a BAC-based infectious cDNA clone with the T25I mutation in nsp5 to study its effect on viral growth. The recombinant virus was generated from the supernatant of the transfected cells. The effect of the T25I mutation on viral growth was assessed using interferon-incompetent VeroE6/hTMPRSS2 cells. These cells were inoculated with the recombinant virus at a multiplicity of infection (MOI) of 0.001, and the levels of infectious virus in the supernatant were measured using TCID_50_ at 12, 24, 36, and 48 h post-infection. The results indicated that the amount of infectious virus and N gene expression in cells infected with rSARS2-QHmusX-T25I were similar to those in cells infected with rSARS2-QHmusX-WT (Fig 3A and 3C).

In interferon-competent 293T/hACE2 cells, the expression of the N gene in T25I virus-infected cells was significantly reduced at 24 and 36 h post-infection compared to that in WT virus-infected cells. Additionally, the number of T25I infectious virus particles in the supernatant was reduced at 48 h post-infection (Fig 3B and 3D). These findings suggest that the nsp5 T25 may influence viral growth in cultured cells in a cell-dependent manner.

### T25I mutation in nsp5 reduces viral replication in mouse lungs

**Fig 4:**
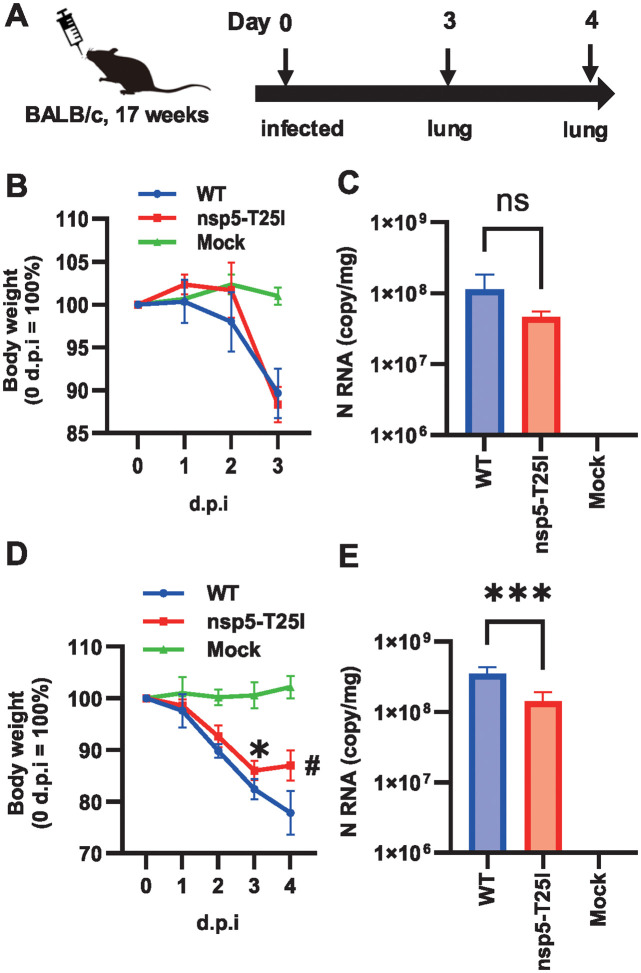
Effect of nsp5 T25I mutation on SARS-CoV-2 proliferation in mouse lung tissue. (A) Schematic of the infection experiments. Changes in body weight after virus inoculation. Day 3 after inoculation, n = 3 (B). Day 4 after infection, n = 5 (D). Expression of the viral N gene in mouse lung tissues. Day 3 after inoculation, Values represent means ± SD of three samples. (C). Day 4 after infection, Values represent means ± SD of five samples. (E). ns, no statistically significant difference; d.p.i., days post-infection.*P < 0.05, ***P < 0.001, and ^#^*P* < 0.0001.

To assess the impact of T25I mutations on viral pathogenesis, 17-week-old female BALB/c mice were intranasally inoculated with rSARS2-QHmusX-T25I. Changes in body weight were monitored, and viral RNA levels in the lung tissue were measured using real-time PCR. In the initial experiment, mice inoculated with WT or T25I exhibited a similar body weight reduction of approximately 90% 3 days post-infection (Fig 4B). RNA expression of the N gene in T25I virus-infected lung tissues was slightly lower, but not significantly different, than that in the WT virus-infected tissues (Fig 4C). In a subsequent experiment with a higher viral dose, both the WT and T25I virus-inoculated groups showed 80–90% body weight loss at 3 days post-infection. However, by day 4, the T25I virus-inoculated group recovered its body weight, unlike the WT group (Fig 4D). Additionally, the expression level of the N gene in T25I virus-inoculated lungs was significantly lower than that in WT virus-inoculated lungs at 4 days post-infection (Fig 4E). These findings suggest that nsp5 T25 is implicated in the pathogenesis of SARS-CoV-2-induced pneumonia.

### T25I virus is less pathogenic in mouse lung tissue

**Fig 5:**
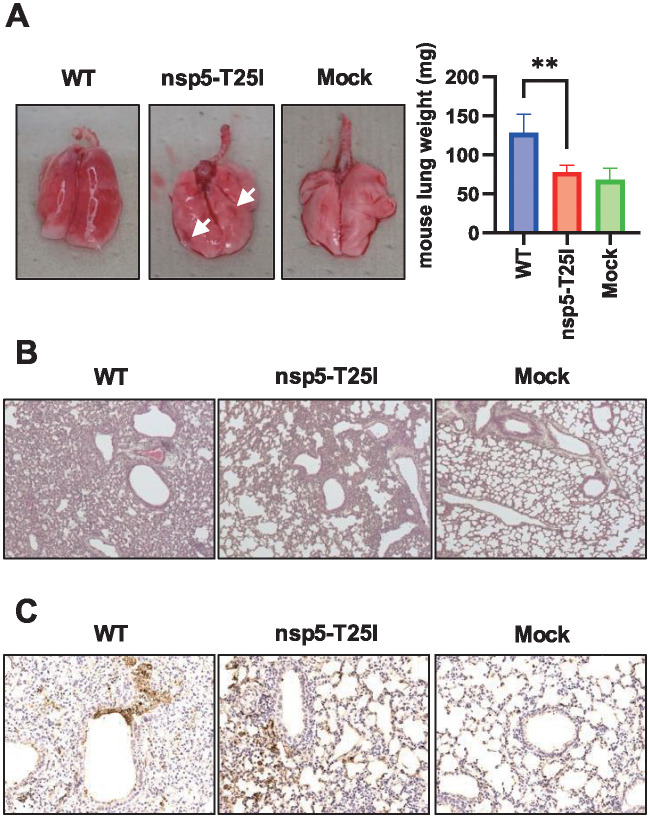
Effect of nsp5 T25I mutation on inflammatory cell infiltration lung tissue. (A) Lung tissues four days after infection. White arrows represent hemorrhagic spots. Lung weight results. Lung weights were measured after autopsy and averaged. Values represent means ±SD of five samples. **P < 0.01. Error bars indicate standard deviation. (A, Graph) Histopathological analysis by HA staining. (B) Histopathological analysis using immunohistochemistry (N antigen). SARS-CoV-2 was detected using an anti-nucleocapsid protein antibody. Brown staining indicates the nucleocapsid (N antigens) of SARS-CoV-2 (C).

Histological analysis of T25I virus-infected lungs at 4 days post-infection was then performed. Macroscopic findings showed obvious hemorrhage in almost all areas of WT-infected lungs, while there was less hemorrhage in T25I-infected lungs (Fig 5A). This indicates that the degree of lung damage caused by T25I is low. In fact, when the weight of infected lungs was measured, the weight of WT-infected lungs was significantly increased compared to that of non-infected lungs (Fig 5A, right graph). Conversely, the weight of T25I-infected lungs was reduced to the same level as that of non-infected lungs (Fig 5A, right graph). This indicated that the degree of inflammatory cell infiltration was at least mild in T25I-infected lungs. In fact, HE staining showed less inflammatory cell infiltration and lung tissue disruption in T25I-infected lung tissue than in WT-infected lung tissue (Fig 5B). In addition, N-antibody-positive cells were more restricted in T25I-infected lung tissue than in WT-infected lung tissue (Fig 5C). These results suggest that T25I virus is less virulent in the mouse lung.

## Discussion

Several studies have reported the importance of the protease activity of SARS-CoV-2 nsp5 in viral replication and the development of viral protease inhibitors [37–39]. Although many studies have used recombinant nsp5 to investigate the effect of candidate protease inhibitors, the correlation between the protease activity of nsp5 and viral replication in SARS-CoV-2 infected cells is still unclear.

Several reports have revealed functional and structural similarities between SARS-CoV-1 nsp5 and SARS-CoV-2 nsp5; both have a molecular weight of approximately 34 kDa and consist of 306 amino acid residues [40, 41]. The main protease domain is formed by several amino acids, including T24, T25, T26, L27, M49, F140, L141, N142, G143, S144, C145, H163, H164, M165, E166, L167, P168, H172, D187, R188, T190, and A191 [14]. nsp5 consists of three domains (I, II, and III), and its substrate-binding pocket is located between domains I and II. Four subsites, S’, S1, S2, and S4, are well defined and can accommodate the P’, P1, P2, and P4 positions in the cleavage substrate, respectively. The S’ binding pocket consists of T25, L27, H41, and C145. The structure of each of these four subsites is critical for protease activity [14]. In fact, our split-luciferase reporter system showed that the T25I substitution reduced nsp5 protease activity in transfected cells.

Mutations in M49, one of the amino acids in S2, also reduced luciferase activity, although to a lesser extent than mutations in T25I [14, 33]. Our results indicate that T25 is important for protease activity. Furthermore, we generated recombinant viruses with the T25I mutation and found that, contrary to expectations, the T25I virus grew in VeroE6/hTMPRSS2 cells as well as the WT virus. This indicates that the T25I mutation attenuates protease activity, but does not significantly affect viral replication. In contrast, the T25 recombinant virus had reduced viral replication in 293T/hACE2 cells, which are IFN-competent cells, compared to the WT. This suggests that the T25I mutation may affect interactions with host factors, such as IFN. In fact, SARS-CoV-2 nsp5 has been reported to suppress the innate immune system via enzymatic activity [42, 43]. Indeed, the mouse strain acclimated to the T25I recombinant virus showed reduced lung growth and regained body weight compared with those infected with WT. Compared to the WT virus, the T25I virus showed a reduced degree of inflammatory cell infiltration and lung tissue damage. This suggests that nsp5 T25 may be involved in the virulence or severity of the disease caused by SARS-CoV-2.

Several studies have reported the relationship between SARS-CoV-2 nsp5 and host factors [28, 44–47]. NEMO, an important factor in the NF-kB pathway, is cleaved by the nsp5 protease [20, 23]. The fact that T25I-inoculated mice showed reduced viral replication and weight recovery suggests that the T25I virus may be less efficient at cleaving NEMO, resulting in the activation of the NF-kB pathway and reduced viral replication in lung tissue. We need to confirm whether the cleavage of NEMO by nsp5 is abolished by the T25I mutation. In addition to NEMO, nsp5 is known to inhibit other important functions of RIG-1 in the innate immune system [28, 42, 43]. nsp5 inhibits the formation of stress granules and interaction of RIG-1 with MAVS [43]. If the mutation in nsp5 does not disrupt the interaction between RIG-1 and MAVS, the innate immune system is expected to eliminate SARS-CoV-2. The reduced growth and attenuated virulence of the recombinant T25I virus in the lungs may be due to the preserved interaction between RIG-1 and MAVS.

The impact of the T25I mutation on enzyme activity was found to be approximately 50% compared to the activity of the catalytic mutant C145A. This could be attributed to the impact of the T25I mutation on S1 structure, particularly involving T25 and its associated side chains, L27, H41, and C145, which collectively constitute S1 through its side chain [14]. The T25I mutation likely caused a minor modification to S1’s structure. The T25I mutation caused a mere 50% reduction in enzyme activity, resulting in minimal changes to viral replication in cultured Vero E6 cells. Conversely, as shown in Fig 4D, the T25I virus inoculation group tended to recover weight by day 4, indicating that the observed effect was not solely attributable to nsp5’s enzymatic activity. It is plausible that the catalytic mutant, nsp5-C145A virus, fails to replicate during the early stages of viral infection, making it impossible to recover the recombinant virus. Conversely, our nsp5-T25I virus could prove a useful tool for investigating novel functions of nsp5, given that the T25I mutation, which reduces enzymatic activity of nsp5 by 50%, allowed for the recovery of an infectious virus.

## Supporting information

S1 Fig Original Blots image, an unprocessed version in Fig 1B and 1D (B: anti-Flag and C: anti-actin). (PDF)

S2 Fig Original Blots image, an unprocessed version in Fig 2D (top: anti-Flag and bottom: anti-actin). (PDF)

S3 Fig Key resources table. (PDF)
