# Supplementary material for: Amino acid T25 in the substrate-binding domain of SARS-CoV-2 nsp5 is involved in viral replication in the mouse lung
Source: PLoS One. 2024 Dec 6;19(12):e0312800. doi: 10.1371/journal.pone.0312800 (PMC11623800; doi:10.1371/journal.pone.0312800)
Supplement: S1 Fig — (PDF) [file pone.0312800.s001.pdf]

Supplementary Materials

Amino acid T25 in the substrate-binding domain of SARS CoV-2 nsp5 is involved in viral replication in the mouse lung

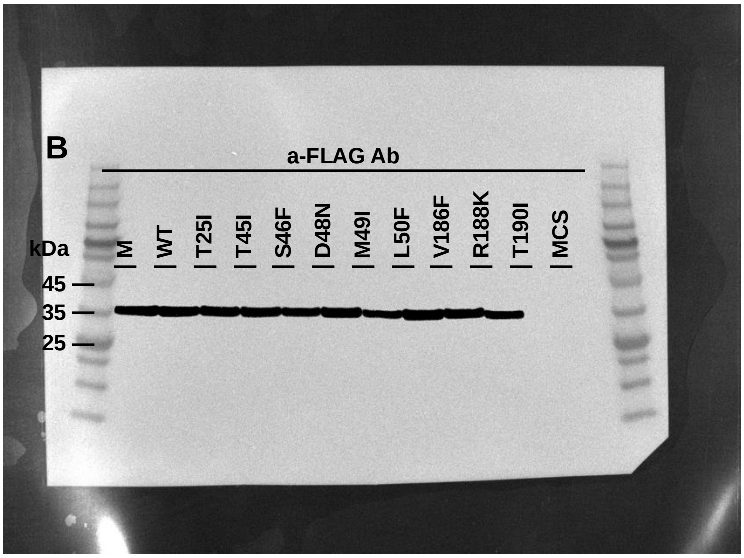

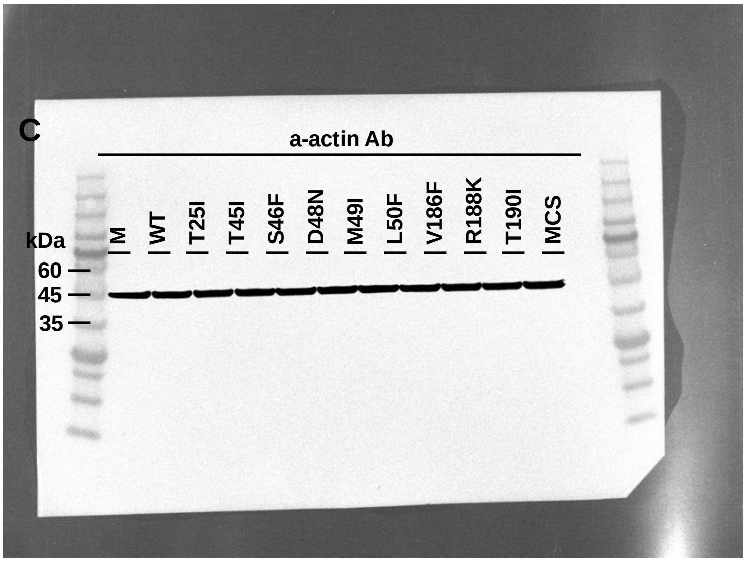

Supplementary Figure 1. Original Blots image, an unprocessed version in Figure 1B and 1D (B: anti-Flag and C: anti-actin)
