# Supplementary material for: Amino acid T25 in the substrate-binding domain of SARS-CoV-2 nsp5 is involved in viral replication in the mouse lung
Source: PLoS One. 2024 Dec 6;19(12):e0312800. doi: 10.1371/journal.pone.0312800 (PMC11623800; doi:10.1371/journal.pone.0312800)
Supplement: S3 Fig — (PDF) [file pone.0312800.s003.pdf]

Key resources table

REAGENT or RESOURCE	SOURCE	IDENTIFIER
Antibodies		
Anti-DDDDK(FLAG)-tag mAb	MBL	Clone FLA-1
Anti-Rabbit IgG (H+L), highly cross-adsorbed, CF™ 488A antibody	Sigma-Aldrich	Cat#SAB4600045
Virus strains		
SARS-CoV-2	NIID	WK-521 strain
SARS-CoV-2	Iwata-Yoshikawa *et al*, 2022	QHmusX strain
Chemicals and critical commercial assays		
Dulbecco's modified minimum essential medium	Nacalai Tesque	Cat#08458-16
Fetal bovine serum	Biosera	Cat#FB-1285
TRIzol™ Reagent	Thermo Fisher Scientific	Cat#15596026
PureLink RNA Mini kit	Thermo Fisher Scientific	Cat#12183018A
THUNDERBIRD Probe One-step qRT-PCR Kit	TOYOBO	Cat#QRZ-101
ReverTraACE qPCR RT Master Mix	TOYOBO	Cat#FSQ-201
THUNDERBIRD SYBR qPCR Mix	TOYOBO	Cat#QPS-201
SuperScript IV	Thermo Fisher Scientific	Cat#18090010
QIAamp viral RNA Mini Kit	QIAGEN	Cat#52904
10% formalin neutral buffer solution	Nacalai Tesque	Cat#37152-51
Mayer's hematoxylin	Muto Pure Chemicals	Cat#30002
1% eosin Y solution	FUJIFILM	Cat#051-0615
KOD One	TOYOBO	Cat#KMM-101
Gibson Assembly Ultra kits	Telesis Bio	Cat#GA1200
TransIT-LT1	Mirus	Cat# MIR2304
4% paraformaldehyde	Nacalai Tesque	Cat#09154-85
Experimental models: Cell lines		
293T/hACE2cells	This paper	N/A
VeroE6/hTMPRSS2 cells	Matsuyama et al, 2020	N/A
Experimental models: mice		
BALB/cCrSlc mice	Japan SLC	N/A
Recombinant DNA		
pBAC-SARS2-QHmusX-WT	This paper	N/A
pBAC-SARS2-QHmusX-T25I	This paper	N/A
pGlo-VTFQS	This paper	N/A
pCAGGS-SARS2-nsp5-T25I-FLAG, pCAGGS-SARS2-nsp5-T45I-FLAG, pCAGGS-SARS2-nsp5-S46F-FLAG, pCAGGS-SARS2-nsp5-D48N-FLAG, pCAGGS-SARS2-nsp5-M49I-FLAG, pCAGGS-SARS2-nsp5-L50F-FLAG, pCAGGS-SARS2-nsp5-V186F-FLAG, pCAGGS-SARS2-nsp5-R188K-FLAG, and pCAGGS-SARS2-nsp5-T190I-FLAG.	This paper	N/A
Software and algorithms		
Prism 10	GraphPad	N/A
